# A study of silicon and germanium-based molecules in terms of solar cell devices performance

**DOI:** 10.55730/1300-0527.3464

**Published:** 2022-06-13

**Authors:** Emine TANIŞ

**Affiliations:** Department of Electrical Electronics Engineering, Faculty of Engineering and Architecture Kırşehir Ahi Evran University, Kırşehir, Turkey

**Keywords:** Reorganization energy, charge transfer rate, silole, germole, DFT, solar cell devices

## Abstract

Photovoltaic energy sources are increasingly in demand due to the cost of petroleum fuels and concerns about carbon emissions. For this reason, it is important to determine the photovoltaic properties of the compounds that are thought to be suitable for these energy sources. Here, 1,1,2,3,4,5-Hexaphenyl-1H-silole (HPS) and 1,1,2,3,4,5-Hexaphenyl-1H-germole (HPG) compounds that are thought to have excellent photovoltaic properties, electronic and charge transport properties were investigated experimentally and theoretically. The total energies, absorption spectra, Fermi energy (E_fl_) and work function (φ), maximum open circuit voltage (V_OC_), reorganization energies (λ_e_ and λ_h_), frontier molecular orbital (HOMO and LUMO), the ionization potentials (IPs) and electron affinities (EAs), effective transfer integrals (V_e_ and V_h_), charge transfer rates (W_e_ and W_h_), molecular electrostatic potential (MEP) surface analysis and Natural Bond Orbital (NBO) analysis were determined and the suitability of the results for photovoltaic solar cell devices was interpreted in detail. The absorbance spectra of the HPS and HPG were experimentally examined and compared to the theoretical results. It can be concluded that HPS and HPG would contribute to the application areas of more effective solar cells with determined properties.

## 1. Introduction

Solar cells have become a very popular energy source in recent years due to the increasing energy demand. This energy source, obtained via photovoltaic technology, is the form of solar energy converted into electrical energy. It is considered that low-cost and nature-friendly solar energy has great potential to contribute to the solution of energy demand [1,2]. High-performance photovoltaic solar cells are the only way to obtain solar energy with the maximum performance [3,4]. Therefore, it is crucial to pay attention to the materials that will improve the performance of solar cells. π-conjugated organic molecules have common usage areas such as organic photovoltaic (OPV) devices, organic field effect transistors (OFETs) [5–7], due to their favorable molecular orbital energies and donor-acceptor structures. In this context, HPS and HPG molecules based on silicon and germanium are examples of π-conjugated organic compounds.

In the literature, there are studies on the reaction chemistry of many molecules containing silicon and germanium [8–12]. Organic/inorganic silicon-based solar cells are candidate materials utilized to improve the performance of heterojunction devices that are employed to increase the energy produced by different electrical devices such as solar cells and lasers [13,14]. Despite its many disadvantages, silicon solar cells are the most widely used photovoltaic technology in space and terrestrial fields [1]. Similarly, some germanium-based compounds are suitable molecules for photovoltaic devices and plastic electronics due to their photophysical properties [15–19]. Faustov et al. [20] reported that the stability and electron paramagnetic resonance (EPR) parameters depended on the conformation of phenyl groups in cyclopentadiene, silole, germole, 1,2,3,4-Tetraphenylcyclopenta-1,3-diene, 2,3,4,5-tetraphenylsubstituted structures, and their radical anions by using ab anitio and DFT methods. Zhan and coworkers [21] evaluated the electron affinities of 1,1-Diaryl-2,3,4,5-tetraphenylsiloles molecules, which are silole-derived molecules, with experimental and theoretical methods. Zhang et al. [22] determined the aggregation emission behaviors using the photophysical properties of HPS by quantum mechanical and molecular mechanical methods. Weijie and colleagues [23] suggested that the HPS nanobeads synthesized hold promise for future biological applications with their excellent emission stability. Ito et al. [24] investigated the phase transformation during evaporative crystallization of HPS with different fluorescent colors using aggregation-induced emission (AIE). Tang et al. [25] synthesized a series of molecules including HPS and its derivatives and determined their photoluminescence properties. However, according to our knowledge, there is no study in which the photovoltaic parameters of HPS and HGS materials that affect the performance of photovoltaic solar cells are calculated in detail in the literature. In addition, there is not yet any study on HPG molecule.

The density functional theory (DFT) and time-dependent (TD)-DFT calculations have been extensively used to investigate the electronic and photovoltaic properties of materials [26,27]. Such theoretical studies are important to save time and money and optimize their experimental procedures. The optimization process starts with the determination of the most suitable geometry where the molecule does not have a negative vibration frequency and continues until the comparison with the experimental results. Similarly, reliable results have been obtained using the DFT and Amsterdam density functional (ADF) methods while assessing the suitability of various structures in terms of solar cells and optoelectronics [28–31]. These studies have also revealed the electronic, optical, and charge transfer properties of molecules in detail.

In the current study, photovoltaic and charge transfer parameters were calculated by DFT and ADF methods to determine the performance of HPS and HPG molecules in terms of photovoltaic solar cells. Considering that the relationship between charge transfer processes and absorbance spectrum may be important in structures with π-conjugated systems such as HPS and HPG, experimental absorbance spectrum results were obtained and compared with theoretical results. In addition, MEP and NBO analyses were performed to determine the reactive sites and charge transfer properties of HPS and HGS molecules. With the results obtained, the suitability of the molecules for solar cells was evaluated in detail.

## 2. Methods

All calculations of the studied molecules were performed using the Gaussian 09 [32] and Amsterdam density functional (ADF2019) [33] programs. Electronic and photovoltaic calculations of HPS and HPG molecules were performed using the TD-DFT/B3LYP/6-311++G(d,p) level of theory.

ADF is a successful computational chemistry software for calculating molecules in terms of structure, electronics, optics, and more [34–36]. Firstly, the dimer structures of HPS and HPG were optimized in the ADF program, and then, the charge transfer properties were calculated.

Charge transfer rates (W) of HPS and HPG are found by the following equation [37–40], known as the Marcus-Hush equation.


(1) $$W=\frac{V^{2}}{\hbar }{\left(\frac{\pi }{\lambda k_{B}T}\right)}^{1/2}\;\text{exp }\left(-\frac{\lambda }{4k_{B}T}\right)$$

where λ is the reorganization energy, T is the temperature, V is the effective charge transfer integral, and ħ and k_B_ are the Planck and Boltzmann constants, respectively.

V_ij_ representing the electronic coupling between intermolecular; depending on the spatial overlap (S_ij_), charge transfer integrals (J_ij_), and site energies (e_i(j)_), respectively,


(2) $$V(ij)=\frac{J_{ij}-S_{ij}(e_{i}+e_{j})/2}{1-S_{ij}^{2}}$$

The reorganization energy related to the charge transport process in organic solids can be defined in two ways which are the normal mode analysis method and the four-point approximation. The normal mode analysis method divides the total relaxation energy by the contributions from each vibration mode. In the four-point approximation, λ can be represented as in equation 3. The total reorganization energy is the sum of the internal reorganization energies arising from intermolecular vibration and external reorganization energies created by the polarization of the surrounding environment. The external reorganization energy of a few tenths of an electron volt is a very small value [41,42]. Therefore, the internal reorganization energies created by an electron, or a hole have been taken into account in the current paper. Electron (or hole) reorganization energies, λ_e_ (or λ_h_) can be written as follows:


(3) $$\begin{array}{l} \lambda_{h}=\lambda_{1}+\lambda_{2}=[E^{+}(g^{0})-E^{+}(g^{+})]+[E^{0}(g^{+})-E^{0}(g^{0})]\\ =[(E^{+}(g^{0})-E^{0}(g^{0})]-[(E^{+}(g^{+})-E^{0}(g^{+}))] \end{array}$$

and


(4) $$\begin{array}{l} \lambda_{e}=\lambda_{3}+\lambda_{4}\\ =[E^{0}(g^{-})-E^{0}(g^{0})]+[E^{-}(g^{0})-E^{-}(g^{-})]\\ =[E^{0}(g^{-})-E^{-}(g^{-})]-[E^{0}(g^{0})-E^{-}(g^{0})] \end{array}$$

Here, E^0^(g^0^) is the energy of the neutral calculated with the optimized structure of the neutral molecule. E^+/−^(g ^+/−^) (E^+^/−(g^0^)) is the energy of cation/anion calculated with the optimized structure of the cation/anion (neutral) geometry. E^0^(g^+/−^) is the energy of the neutral molecule calculated with the optimized structure of the cation/anion geometry.

The injection processes of holes and electrons, which affect the performance of the devices, are highly dependent on their stability and energy barriers [43]. There are many studies in the literature about the injection ability of molecules depending on ionization potentials (IPa/IPv), HOMOs, LUMOs, and electron affinities (EA_a_/Ea_v_) [44–47]. The IP_v_/IP_a_, Ea_v_/EA_a_ of the studied structures were obtained as follows:


(5) $$IP(v)=E^{+}(g^{0})-E^{0}(g^{0})$$

(6) $$IP(a)=E^{+}(g^{+})-E^{0}(g^{0})$$

(7) $$EA(v)=E^{0}(g^{0})-E^{-}(g^{0})$$

(8) $$EA(a)=E^{0}(g^{0})-E^{-}(g^{-})$$

The open-circuit voltage (V_OC_) is defined as the voltage in the zero current state and is closely related to charge recombination [48]. The open-circuit voltage (V_OC_) of photovoltaic molecules can be calculated by [49]:


(9) $${\text{V}}_{\text{OC}}=\;|E_{HOMO}(Donor)|-|E_{LUMO}(Acceptor)|-0.3$$

Here, both HPS and HGS molecules were used as a donor, and a fullerene-derived molecule, PC_60_BM, was used as an acceptor. PC_60_BM is a molecule with an efficiency higher than 11% in conventional organic solar cells, and it is widely used in calculations [49–51].

Calculation of fermi energy level (E_fl_) and work function (φ) is important for photoelectronic applications. E_fl_ and φ were calculated using the following equations:


(10) $${\text{E}}_{\text{fl}}=({\text{E}}_{\text{HOMO}}+{\text{E}}_{\text{LUMO}})/2$$

(11) $$\varphi ={\text{E}}_{\text{vac}}-{\text{E}}_{\text{fl}}$$

Here, the vacuum energy level (E_vac_) is the energy barrier preventing the electron from being completely separated from the material [52].

## 3. Experimental

HPS and HPG molecules were purchased from Sigma-Aldrich Co. LLC. in solid form with a purity of above 97%. UV-vis absorbance spectra were obtained in chloroform solvent at room temperature using a UV-1800 spectrophotometer (Shimadzu).

## 4. Results and discussion

### 4.1. Geometric structures and electronic properties

**Figure 1:**
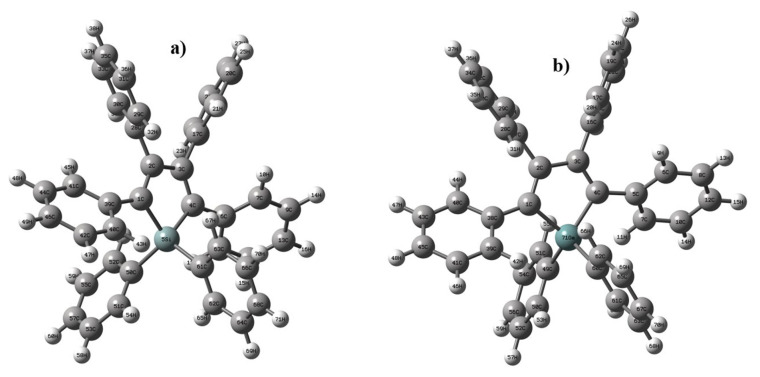
Optimized molecular structures of a) HPS and b) HPG.

The monomer structures of HPS and HPG optimized at the DFT/B3LYP/6-311++G(d,p) level of theory are portrayed in Figure 1 (a, b). The total energies of HPS and HPG were calculated to find the most stable structure. The most stable molecule is the one with the lowest energy [53]. The energy of the HPS molecule was −1832.21106368 a.u and the energy of the HPG molecule was −3619.68363740 a.u. This result showed that the HPG molecule was more stable than HPS.

Determining the interaction between a molecule and beam is vital to understand its electronics and to design new photovoltaic devices [54]. The computed absorption wavelengths in chloroform solvent by using TD-DFT/B3LYP/6-311++G(d,p) level theory are listed in Table 1. The computed absorption wavelength of the HPS was at 346 nm (3.59 eV). The major transition was from H→L. The second and third peaks were calculated at 284 (4.36 eV) and 266 nm (4.66 eV) with the transition from H-1→L and H-2→L, respectively. It was determined that the maximum absorbance peaks of HPG had H→L, H-1→L, and H-2→L transitions at wavelengths of 354 nm (3.50 eV), 281 nm (4.41 eV) and 266 nm (4.66 eV), respectively.

**Figure 2:**
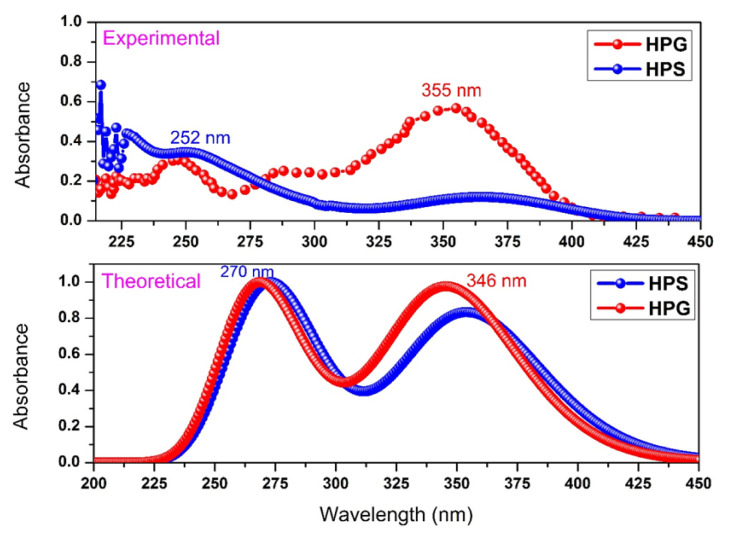
Absorbance spectra curves of HPS and HPG.

The experimental and theoretical absorbance spectra curve of HPS and HPG are shown in Figure 2. Here, it was observed that HPS gave a maximum absorbance peak at 252 nm (4.92 eV) and HPG at 355 nm (3.49 eV), experimentally. Tang and his colleagues [25] observed the experimental maximum absorbance spectrum in chloroform solvent of HPS at about 250 nm. This result is in good agreement with our result. Zhang et al. [22], measured the experimental absorbance spectrum of HPS as approximately 350 nm. The small difference between this result and our measurement could be due to the difference in phases while in our experiment HPS was in chloroform solution, it was in a solid state in the work done by Zhang et al. [22]. Wu et al. [23] also measured the experimental maximum absorbance spectrum of HPS in toluene solvent as approximately 450 nm.

From the theoretical absorbance spectra calculated by the TD-DFT method in Figure 2, it was seen that HPS and HPG molecules had a maximum absorbance spectrum of 270 nm (4.59 eV) and 346 nm (3.58 eV), respectively. Therefore, it can be concluded that they are compatible with both experimental results and literature [25]. In addition, the absorbance spectra of both are in the near ultraviolet region.

**Figure 3:**
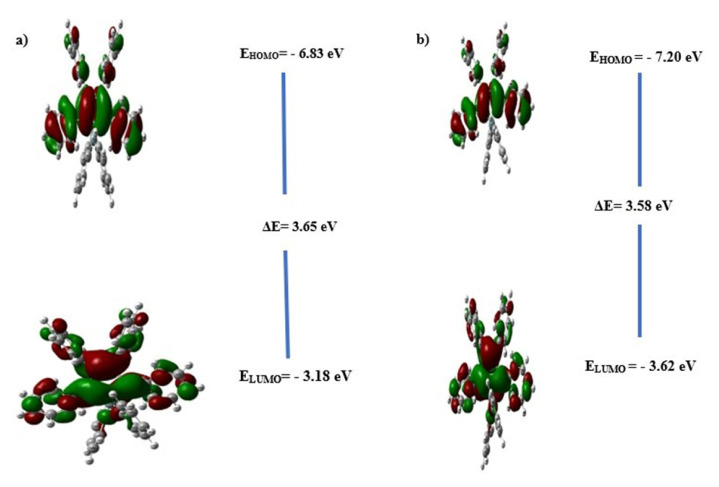
Frontier orbital contour plot a) HPS and b) HPG.

Frontier molecular orbitals called HOMO, LUMO and the energy difference between these orbitals are important in terms of charge transfer. Figure 3 (a, b) shows the contour plots of the HOMO and LUMO molecular orbitals and the energy values of these orbitals for the studied molecules. As seen in Figure 3 (a, b), the phenyl rings attached to the silole and germanium atoms in both molecules do not contribute to the HOMO and LUMO orbitals. As seen in Table 2, the energies of the HOMO orbitals were calculated as −7.20 eV for HPG and −6.83 eV for HPS. A molecule with a large HOMO energy level means that it has a more favorable hole transport and thus a hole transfer integral [55]. Therefore, the HOMO of HPS was higher in terms of hole-creating potential. The lower the LUMO energy level of a molecule, the higher its electron injection ability and the more stable the injected electron [56]. The energy LUMO orbital of HPG’s (−3.62 eV) was lower than that of HPS (−3.18 eV). Therefore, it indicated a higher electron injection ability of HPG.

The difference between the HOMO and LUMO energy levels, called the electrochemical band gap, and the excitation energy for the transitions between the vertical bands, called the optical band gap, are different energies [57]. The optical band gap is obtained by taking the difference between the vertically excited energy levels from the calculation of the excited energy levels. Here, using the TD-DFT method, electrochemical band gap values of HPG and HPS were calculated as 3.58 eV and 3.65 eV, and optical band gap values were calculated as 3.58 eV and 3.50 eV, respectively (Table 1).

The molecule whose V_OC_ value is closest to the LUMO energy level value is more suitable for efficient photovoltaic devices. For efficient photovoltaic devices, electrons must be able to easily injected from the LUMO into the conduction band of semiconductors [58]. Furthermore, the LUMO of the donor must be higher than the LUMO of the acceptor. As seen in Table 2, the V_OC_ value of HPG was more suitable for photovoltaic device technology and can perform electron transfer with smaller energy (0.852 eV). The work function, which determines the minimum energy required for an electron lifted from the Fermi level to the vacuum level, limits the potential barrier for electron emission.

### 4.2. Reorganizational energies, ionization potentials and electron affinities

The calculated λ_e_/λ_h_, IP_a_/IP_v_, and EA_a_/Ea_v_ values of HPS and HPG molecules are tabulated in Table 3. These calculated values are factors affecting the performance of solar cells. For example, the mobility of electrons and holes is closely related to reorganizational energy. The λ_e_ value of HPG was calculated to be smaller than that of HPS, and the λ_h_ value of HPS was calculated to be smaller than that of HGS. Therefore, HPG had potential to be a perfect electron transfer material, and HPS could be excellent hole transfer material.

It is known that the reorganization energy is not sufficient to determine the charge transfer of material. Besides, ionization potentials (IPs), electron affinities (EA’s), dipole moments and transfer integral values were calculated and interpreted using computational chemistry methods. Magnitudes such as EA and IP significantly affect the energy threshold available for the injection of holes and electrons in a material. The bigger EA and smaller IP mean better electron and hole transport [59]. In other words, it can be said that n-channel molecules have a higher EA value and p-channel molecules have a smaller IP value [60]. Table 3 shows all EA_a/v_ and IP_a/v_ values of HPS and HPG molecules. The EA_a/v_ and IP_a/v_ values of HPG were 2.231/1.986 eV and 6.639/6.884 eV, respectively. Similarly, the EA_a/v_ and IP_a/v_ values of HPS were 0.816/0.544eV and 4.687/4.959 eV, respectively. Consequently, it can be said that HPG is a good electron transport material and, HPS is the best hole transport material.

### 4.3. Dipole moment

Another factor that affects the performance of solar cells is the dipole moment. The dipole moment plays an important role in the production of solar cells as it affects the solubility of a molecule in any solvent [61]. A molecule with a dipole moment is known to have high solubility in chloroform, an organic solvent. For this reason, dipole moments of the studied materials were calculated by using a B3LYP/6-311++G(d, p) basis set in chloroform solvent. As seen in Table 3, HPG had a larger dipole than HPS. Therefore, HPG can self-assemble in the chloroform solvent, indicating that it has greater charge transfer than HPS.

### 4.4. Effective transfer integrals and charge transfer rates

**Figure 4:**
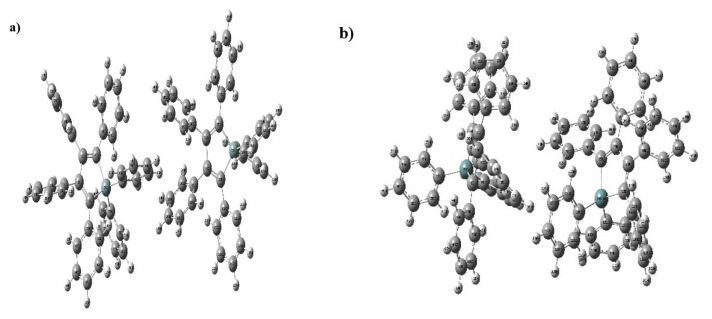
Optimized structure of a) HPS dimer and b) HPG dimer.

The effective transfer integrals V represents the strength of the electronic coupling between two neighboring molecules i and j and may vary depending on the geometry of the dimer. Besides, the charge transfer must be anisotropic [39,40,62,63]. Since the interactions between the π-conjugate coupling, which facilitates charge transport between neighboring compounds, are strong, the molecules forming the dimer were considered in parallel while creating the anisotropic geometry. Optimized dimer structures obtained by using parallel dimer geometries for HPG and HPS are given in Figure 4 and transfer integrals belonging to these geometries are tabulated in Table 4. In general, a material with a large transfer integral will also have a large charge transfer. As seen in Table 4, HPG had a higher electron transfer integral (absolute) than HPS. Moreover, the hole transfer integral of HPS was much higher than HPG. Thus, it can be said that HPS is a good candidate for being a hole transfer material.

Charge transfer rates (W_electron_ and W_hole_) were calculated using equation 1 for HPS and HPG in parallel geometry at room temperature and are tabulated in Table 4. HPG molecule had a high electron transfer rate (530 × 10^9^ s^−1^). On the other hand, the HPS molecule had a large hole transfer rate (140 × 10^9^ s^−1^).

### 4.5. Molecular electrostatic potential (MEP) analysis

**Figure 5:**
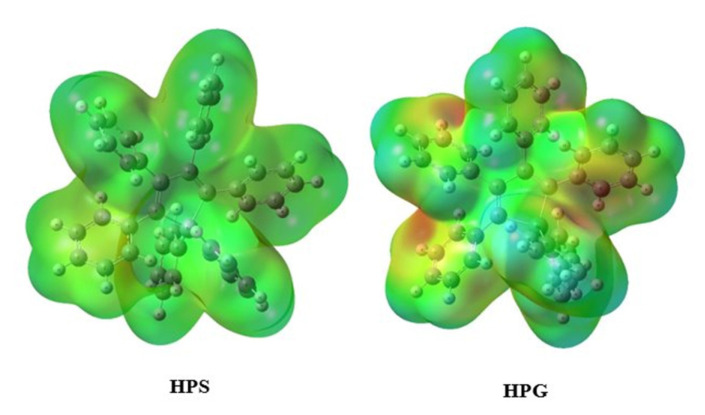
MEP surfaces of the HPS and HPG.

The electrostatic molecular potential (MEP) surface, which helps understand the physicochemical structure of a molecule, provides a three-dimensional graphical representation of the molecule depending on its electron density [64]. In this visual presentation, red (negative) regions show electrophilic reactivity which is electron-rich regions, and blue (positive) regions show nucleophilic reactivity which is electron-accepting regions, and green regions show neutral region which is zero potential. MEP studies were carried out using the B3LYP/6-311++G(d,p) basis set and are presented in Figure 5. The color range of the surface was between −9.131e^−2^ and 9.131e^−2^, −8.012e^−2^ and 8.012e^−2^ for HPS and HPG, respectively. It can be seen from Figure 5 that HPG had more red color intensity showing that HPG is more reactive and electrophilic than HPS.

### 4.6. NBO analysis

NBO analysis is an efficient method for understanding electron transfer between Lewis (donor (i)) and non-Lewis (acceptor (j)) orbitals [65]. The stabilization energy (E^(2)^), which is a measure of electron transfer by NBO analysis, is estimated by the following equation:


(12) $$E^{\left(2\right)}=\Delta E_{ij}=q_{i}\frac{F{\left(i,j\right)}^{2}}{{ɛ}_{j}-{ɛ}_{i}}$$

where F(i,j), ɛ_j_ and ɛ_i_,q_i_ – were diagonal NBO Fock matrix element, the diagonal elements and the donor orbital occupancy, respectively. NBO analysis was carried out by using NBO 3.1 program [66] included in the Gaussian 09 software. Intramolecular interactions with charge transfer for the most significant stabilization energies E(2), obtained from NBO calculations, are presented in Table 5 and Table 6. The larger the value of E(2), the greater the degree of conjugation or charge transfer of the entire system [67,68]. Compared the calculated NBO results in Table 1 and Table 2 indicated that the stabilization energies of HPG were greater than HPS. For example, the most interesting stabilization energies for HPG were calculated as 21.96 kcal/mol, 19.91 kcal/mol, 25.96 kcal/mol, 41.42 kcal/mol and 74.63 kcal/mol, respectively, which were associated with the donor-acceptor transition of C1-C2→C3-C4, C1-Ge71→C2-C27, C3-C4→C1-C2, C5-C7→C10-C12. On the other side, the most interesting stabilization energies for HPS were calculated as 12.04 kcal/mol, 10.32 kcal/mol, 21.40 kcal/mol, 24.83 kcal/mol, 43.30 kcal/mol, respectively, which are associated with the donor-acceptor transition of C3-C4→C1-C2, C1-Si5→C2-C28, C6-C8→C11-C13, C17-C19→C3-C4, C39-C40→C1-C40. As a result, it was seen that the interaction in HPG is stronger than in HPS.

## 5. Conclusion

In the present study, advanced quantum theoretical calculations were used to examine the charge transfer properties, photovoltaic and electronic properties of HPS and HPG. The absorbance spectra obtained by the experimental analysis method were found to be quite compatible with the calculated absorbance spectra. Structurally, HPG was found to be more stable than HPS due to its lower energy. The reorganization energies and Marcus-Hush theory indicated that HPG can be used as electron transport material and HPS can be used as hole transport material. Through the MEP map, it can be said that HPG was more electrophilic than HPS. These results can also be seen from the values of effective transfer integrals and charge transfer rates. In terms of the calculated work function and open-circuit voltage, it was found that HPG is more suitable for photovoltaic properties. In all the properties obtained, it can be said that the conjugation in the ring was an important factor in the transition from silicon to germanium. Finally, it was determined that HPG was a better candidate than HPS for solar cell devices due to its absorbance spectra, electrochemical, optical band gaps, photovoltaic properties and high E(2) stabilization energy. It is hoped that the results will provide further insight into experimental studies on the design of higher-performance photovoltaic devices.

## Figures and Tables

**Table 1:** The computed absorption wavelengths, excitation energies, absorbance, and oscillator strengths.

HPG	λ (nm)	*E (eV)*	*f*	Transition
	354	3.50	0.2131	H→L
	281	4.41	0.1318	H–1→L
	266	4.66	0.1522	H–2→L
HPS	346	3.58	0.2483	H→L
	284	4.36	0.0545	H–1→L
	266	4.66	0.2210	H–2→L

**Table 2:** The HOMO, LUMO, E_gap_, fermi energy level (E_fl_), vacuum energy level (E_vac_) and work function (φ). All energies are in eV unit. V_OC_ values are obtained using the LUMO of PC_60_BM as an acceptor.

Molecules	HOMO	LUMO	E_gap_	E_fl_	E_vac_	φ	V_oc_
HPS	−6.83	−3.18	3.65	−5.005	2.758	2.247	2.936
HPG	−7.20	−3.62	3.58	−5.410	4.558	0.852	3.290

**Table 3:** The calculated reorganization energies (λ_electron_/λ_hole_), vertical and adiabatic ionization potentials (IP_a_/IP_v_), vertical and adiabatic electronic affinities (EA_v_/EA_a_) (in eV).

Molecules	λ_electron_	λ_hole_	IP_a_	IP_v_	EA_a_	EA_v_	Dipole moment (Debye)
HPS	0.417	0.326	4.687	4.959	0.816	0.544	0.0184
HPG	0.244	0.598	6.639	6.884	2.231	1.986	0.1909

**Table 4:** The charge transfer integrals (V_electron_/V_hole_) (in eV) and the charge transfer rates (W_electron_/W_hole_) (in s^−1^).

Molecules	V_electron_	V_hole_	W_electron_	W_hole_
HPS	−0.00034	−0.00207	2.6 × 10^9^	140 × 10^9^
HPG	0.00047	0.00002	530 × 10^9^	5 × 10^9^

**Table 5:** Second order perturbation theory analysis of Fock matrix in NBO basis for HPS.

Donor (i)	Type	ED/e	Acceptor(j)	Type	ED/e	E^(2)a^(KJ mol^−1^)	E(j)-E(i)^b^ (a.u)	F(i.j)^c^ (a.u)
C1–C2	σ	1.96	C1–Si5	σ*	0.03	2.45	0.99	0.044
C1–C2	π	1.96	Si5–C61	π*	0.03	2.59	0.54	0.034
C1–Si5	σ	1.96	C2–C28	σ*	0.01	10.32	0.94	0.088
C1–C39	σ	1.59	C1–C2	π*	0.31	5.16	1.28	0.073
C1–C39	σ	1.59	C1–Si5	σ*	0.35	4.75	0.91	0.036
C2–C3	σ	1.97	C1–C39	σ*	0.03	4.90	1.11	0.066
C2–C28	σ	1.97	C1–C2	σ	0.03	5.35	1.27	0.074
C2–C28	σ	1.97	C1–Si5	σ*	0.01	2.93	0.90	0.046
C3–C4	π	1.98	C1–C2	π*	0.03	12.04	0.32	0.055
C3–C4	π	1.98	Si5–C61	σ*	0.02	2.32	0.54	0.032
C4–Si5	σ	1.97	C3–C17	σ*	0.03	10.32	0.94	0.088
C6–C8	π	1.97	C11–C13	π*	0.01	21.40	0.28	0.069
C7–C9	π	1.98	C11–C13	π*	0.03	20.50	0.28	0.068
C7–H10	σ	1.98	C6–C8	σ*	0.02	4.41	1.07	0.061
C8–H12	σ	1.98	C6–C7	σ*	0.01	4.35	1.08	0.061
C9–C13	σ	1.97	C7–C9	σ*	0.02	3.19	1.28	0.057
C9-H14	σ	1.97	C6–C7	σ*	0.02	4.31	1.08	0.061
C11–C13	π	1.72	C6–C8	π *	0.5	19.98	0.29	0.068
C17–C19	π	1.72	C22–C24	σ*	0.01	21.08	0.28	0.069
C18–C20	π	1.98	C22–C24	π*	0.03	20.62	0.28	0.068
C28–C29	σ	1.71	C31–C35	σ*	0.31	21.12	0.28	0.069
C30–C33	π*	1.72	C28–C29	σ*	0.47	20.47	0.29	0.069
C39–C40	π	1.72	C1–Si5	π*	0.31	1.06	0.51	0.023
C41–C44	π	1.98	C39–C40	π*	0.02	20.07	0.28	0.068
C50–C51	σ	1.98	Si5–C50	σ*	0.02	2.42	0.97	0.043
C51–C53	σ	1.88	Si5–C50	σ*	0.47	3.20	0.97	0.050
C52–C55	π	1.57	C53–C57	π*	0.47	20.86	0.28	0.069
C53–C57	π	1.57	C50–C51	π*	0.35	22.01	0.28	0.070
C61–C62	σ	1.57	Si5–C61	σ*	0.35	2.42	0.97	0.043
C61–C62	σ		C1–Si5	σ*		0.89	0.52	0.021
C61–C62	π		C63–C66	π*		20.70	0.28	0.069
C61–C63	σ		Si5–C61	σ*		2.52	0.97	0.044
C63–C66	π		C64–C68	π*		20.86	0.28	0.069
C29–C32	π		C27–C28	π*		20.52	0.29	0.069
C6	CR(1)		C4–Si5	σ*		0.76	10.31	0.080
C6–C8	π		C3–C4	π^*^		63.30	0.01	0.049
C17–C19	π		C3–C4	π*		24.83	0.02	0.036
C39–C40	π		C1–C2	π*		43.30	0.01	0.049
C39–C40	σ		C1–Si5	σ*		1.41	0.01	0.035

**Table 6:** Second order perturbation theory analysis of Fock matrix in NBO basis for HPG.

Donor (i)	Type	ED/e	Acceptor(j)	Type	ED/e	E^(2)a^(KJ mol^−1^)	E(j)–E(i)^b^ (a.u)	F(i.j)^c^ (a.u)
C1–C2	σ	1.96	C1–C38	σ*	0.03	4.10	1.22	0.063
C1–C2	σ	1.96	C1–Ge71	σ*	0.03	1.69	0.93	0.036
C1–C2	σ	1.96	C2–C3	σ*	0.01	4.03	1.17	0.061
C1–C2	σ	1.59	C2–C27	σ*	0.31	5.02	1.19	0.069
C1–C2	π	1.59	C3–C4	π*	0.35	21.96	0.32	0.055
C1–C2	π	1.97	C38–C39	π*	0.03	6.33	0.31	0.042
C1–C2	π	1.97	C49–Ge71	π*	0.03	2.12	0.48	0.029
C1–C38	σ	1.97	C1–C2	σ*	0.01	4.91	1.29	0.071
C1–Ge71	σ	1.98	C2–C27	σ*	0.03	19.91	0.94	0.087
C1–Ge71	σ	1.98	C38–C40	σ*	0.02	13.06	1.04	0.053
C2–C3	σ	1.97	C1–C38	σ*	0.03	4.88	1.11	0.059
C2–C3	σ	1.97	C16–C18	σ*	0.01	0.97	0.65	0.024
C2–C27	σ	1.98	C1–C2	σ*	0.03	5.47	1.27	0.075
C2–C27	σ	1.98	C2–C3	σ*	0.02	1.90	1.07	0.040
C3–C4	σ	1.98	C2–C3	σ*	0.01	4.03	1.17	0.061
C3–C4	σ	1.97	C3–C16	σ*	0.02	5.02	1.19	0.069
C3–C4	σ	1.97	C4–Ge71	σ*	0.02	1.69	0.93	0.069
C3–C4	π	1.72	C16–C18	π *	0.5	0.67	0.76	0.022
C3–C4	π	1.72	C1–C2	σ*	0.01	25.96	0.32	0.055
C3–C4	σ	1.98	C5–C7	σ*	0.03	6.33	0.31	0.042
C3–C4	σ	1.71	C60–Ge71	σ*	0.31	2.12	0.48	0.029
C3–C16	σ	1.72	C1–C2	σ*	0.47	1.78	1.27	0.043
C4–C5	σ	1.72	C2–C3	σ*	0.31	3.84	1.29	0.058
C4–C5	σ	1.98	C4–Ge71	σ*	0.02	1.02	0.86	0.027
C4–Ge71	σ	1.98	C1–Ge71	σ*	0.02	11.75	0.68	0.031
C4–Ge71	π	1.88	C3–C16	π*	0.47	19.91	0.94	0.078
C5–C6	π	1.57	C3–C4	π*	0.47	0.85	0.73	0.023
C5–C7	π	1.57	C6–C8	π*	0.35	19.03	0.28	0.066
C5–C7	π	1.57	C10–C12	π*	0.35	41.42	0.28	0.069
C6–C8	π		C5–C7	π*		20.03	0.28	0.068
C10–C12	π		C6–C8	π*		19.93	0.29	0.068
C17–C19	π		C16–C18	π*		40.52	0.29	0.069
C21–C23	π		C16–C18	π*		40.45	0.29	0.069
C29–C32	π		C27–C28	π*		30.52	0.29	0.069
C38–C39	π		C1–Ge71	π*		1.28	0.45	0.023
C49–Ge71	σ		C4–Ge71	σ*		2.56	0.72	0.038
C60–C61	π		C1–Ge71	π*		0.77	0.46	0.018
C60–C61	π		C62–C65	π*		20.61	0.28	0.069
C5–C7	π		C3–C4	π*		74.63	0.02	0.051
